# Myths and common misbeliefs about cervical cancer causation among Palestinian women: a national cross-sectional study

**DOI:** 10.1186/s12889-024-17733-5

**Published:** 2024-01-16

**Authors:** Mohamedraed Elshami, Hanan Abukmail, Mariam Thalji, Ibrahim Al-Slaibi, Mohammed Alser, Afnan Radaydeh, Alaa Alfuqaha, Salma Khader, Lana Khatib, Nour Fannoun, Bisan Ahmad, Lina Kassab, Hiba Khrishi, Deniz Elhussaini, Nour Abed, Aya Nammari, Tumodir Abdallah, Zaina Alqudwa, Shahd Idais, Ghaid Tanbouz, Ma’alem Hajajreh, Hala Abu Selmiyh, Zakia Abo-Hajouj, Haya Hebi, Manar Zamel, Refqa Najeeb Skaik, Lama Hammoud, Saba Rjoub, Hadeel Ayesh, Toqa Rjoub, Rawan Zakout, Amany Alser, Shurouq I. Albarqi, Nasser Abu-El-Noor, Bettina Bottcher

**Affiliations:** 1 Division of Surgical Oncology, Department of Surgery, University Hospitals Cleveland Medical Center, 11100 Euclid Avenue, Lakeside 7100, Cleveland, OH USA; 2 Ministry of Health, Gaza, Palestine; 3 Department of Public Health and Primary Care, University of Cambridge, Cambridge, UK; 4 Harvard Medical School, Boston, MA USA; 5 International Medical Corps, Gaza, Palestine; 6 Faculty of Medicine, Al-Quds University, Jerusalem, Palestine; 7 Almakassed Hospital, Jerusalem, Palestine; 8 The United Nations Relief and Works Agency for Palestine Refugees in the Near East (UNRWA), Gaza, Palestine; 9 Faculty of Graduate Studies, An-Najah National University, Nablus, Palestine; 10 Faculty of Medicine, An-Najah National University, Nablus, Palestine; 11 Faculty of Pharmacy, Alazhar University of Gaza, Gaza, Palestine; 12 Faculty of Medicine, Islamic University of Gaza, Gaza, Palestine; 13 Faculty of Dentistry and Dental Surgery, Al-Quds University, Jerusalem, Palestine; 14 Faculty of Medicine, Alazhar University of Gaza, Gaza, Palestine; 15 Hebron Governmental Hospital, Hebron, Palestine; 16 Alia Hospital, Hebron, Palestine; 17 Al-Shiffa Hospital, Gaza, Palestine; 18 Faculty of Nursing, Islamic University of Gaza, Gaza, Palestine

**Keywords:** Cervical cancer, Myths, Mythical causes, Beliefs, Behavioral changes, Palestine

## Abstract

**Background:**

Cervical cancer (CC) myths and beliefs can negatively impact women's preventive behaviors, including vaccination against human papillomavirus and having regular screening tests. This study aimed to examine awareness of Palestinian women about myths related to CC causation and investigated factors associated with good awareness.

**Methods:**

A national cross-sectional study was conducted to recruit adult Palestinian women from hospitals, primary healthcare facilities, and public areas in 11 Palestinian governorates. A translated-into-Arabic version of the Cancer Awareness Measure-Mythical Causes Scale was used to collect data. Awareness level was determined based on the number of CC myths around CC causation recognized to be incorrect: poor (0–4), fair (5–9), and good (10–13).

**Results:**

A total of 7058 questionnaires were included. Myths unrelated to food were more commonly recognized as incorrect compared to those related to food. The most recognized food-unrelated myth was ‘having a physical trauma’ (*n* = 3714, 52.6%), whereas the least recognized was ‘using mobile phones’ (*n* = 2238, 31.7%). The most recognized food-related myth was ‘drinking from plastic bottles’ (*n* = 2708, 38.4%), whereas the least recognized was ‘eating food containing additives’ (*n* = 1118, 15.8%).

Only 575 participants (8.1%) displayed good awareness and promptly recognized at least 10 out of 13 myths around CC causation as incorrect. Factors associated with lower likelihood of displaying good awareness of myths around CC causation included living in the West Bank and Jerusalem, being married, widowed or divorced, knowing someone with cancer, and visiting hospitals or primary healthcare centers.

**Conclusions:**

A very small proportion of Palestinian women recognized 10 or more myths around CC causes as incorrect. Initiatives addressing CC myths are needed in the Palestinian community.

**Supplementary Information:**

The online version contains supplementary material available at 10.1186/s12889-024-17733-5.

## Introduction

Cervical cancer (CC) is a significant cause of cancer-related morbidity and mortality worldwide [1, 2]. A total of 604,000 women were diagnosed with CC in 2020, making it the fourth most commonly diagnosed cancer among females globally [2]. Approximately 85% of CC-related deaths occur in low- and middle-income countries, where the disease burden is noticeably higher in terms of incidence, death, and disability adjusted life years [3, 4]. In Palestine, CC is the third most common gynecological cancer with an age-standardized incidence rate of 2.5 per 100,000 females in 2018 [5]. Reports from Globocan 2020 indicate that the 5-year prevalence of CC is 6.05 per 100,000 female population. In addition, there were 64 Palestinian women diagnosed with CC in 2020, of whom 44 (68.8%) died from their disease [6].

Given the presence of human papillomavirus (HPV) vaccinations, CC is considered a preventable cancer and is highly treatable if detected early by screening programs [7, 8]. While having access to efficient screening and medical care facilities is crucial to preventing and detecting CC, shared myths and sociocultural misconceptions about the disease may impact women's preventive behaviors. A previous study from the United Kingdom concluded that CC mythical causes could include eating food containing additives, using microwave ovens, having a physical trauma, feeling stressed, living near power lines and exposure to electromagnetic frequencies [9]. The way by which women perceive CC risk factors may play a role in prompt risk reduction behaviors [10]. However, behavior modification for true CC risk factors may be less likely to occur if behavioral changes are dominated by myths around CC causes [10, 11].

In Palestine, both HPV vaccination and CC screening program are not part of the national health policy [12]. Previous studies assessing the awareness level of CC risk factors, warning signs, and HPV vaccines demonstrated a lack of knowledge among Palestinian women [13–15]. Women's CC-related risk perceptions and attitudes may contribute to their adoption of healthy behaviors. Such healthy preventive behaviors of CC include vaccination against HPV, getting screened regularly, stopping smoking, and practicing safe sexual contact [16]. Therefore, a thorough understanding of how mythical causes of CC are perceived by Palestinian women is needed as a crucial step in launching educational interventions and promoting CC preventive practices. As a result, this study aimed to (1) examine the recognition of myths around CC causes to be incorrect among Palestinian women (2) make comparisons between women living in the two main areas of Palestine: West Bank and Jerusalem (WBJ) and the Gaza Strip, and (3) examine the factors associated with a better ability to recognize such myths as incorrect.

## Materials and Methods

### Study design and population

A cross-sectional, descriptive design was used in this study. Data were collected from Palestine's main two regions, the Gaza Strip and the WBJ, between the 1st of July 2019 and 31st of March 2020. About 50% of Palestinian women are in their childbearing age [17]. Therefore, adult women (aged over 18 years) were recruited from 11 governorates, out of 16 Palestinian governorates, with four governorates in the Gaza Strip (North of Gaza, Gaza, Middle Zone, and Khanyounis) and seven governorates in the WBJ (Hebron, Nablus, Ramallah, Tulkarm, Bethlehem, Jenin, and Jerusalem). Those governorates were chosen as they covered the majority of the Palestinian Population.

### Sample size calculation

In 2019, the female population aging 15 years or older was 1,534,371 in Palestine [18]. With a type I error rate of 5.0%, a confidence level of 95.0%, and an absolute error of 1.0%, the minimum sample size required to detect a 10% good overall awareness of CC causation myths was 900 participants.

### Sampling methods

Convenience sampling was utilized to recruit eligible women from hospitals, primary healthcare facilities, and public spaces. The public spaces included shopping centers, markets, parks, restaurants, mosques, churches, and transport networks. The data collection sites were distributed across various locations in Palestine to maximize the representativeness of the study cohort for the Palestinian community [13–15, 19–27].

### Inclusion and exclusion criteria

Adult Palestinian women who were visiting one of the data collection sites were eligible. Women having nationalities other than Palestinian, those who were working or pursuing education in a health-related field, those who were attending oncology departments at the time of data collection, and those who were unable to complete the questionnaire were all excluded from the study.

### Measurement tool and data collection

A modified version of the Cancer Awareness Measure-Mythical Causes Scale (CAM-MYCS) was used to collect data [9]. The CAM-MYCS was translated from English to Arabic by two bilingual healthcare professionals, and then back-translated from Arabic to English by two other bilingual healthcare professionals. All these healthcare professionals had relevant expertise in gynecologic oncology, public health, and survey design. Five independent healthcare professionals and researchers evaluated the questionnaire's content validity. Then, a pilot study (n = 130) was carried out in order to evaluate the clarity of the Arabic questionnaire. The final analysis did not include the questionnaires of the pilot study. The questionnaire had an acceptable internal consistency with Cronbach's Alpha of 0.79.

The study questionnaire is provided in supplementary file 1. The questionnaire comprised two sections. The first section described sociodemographic data including age, highest level of education, employment status, monthly income, marital status, knowing someone with cancer, place of residence, having a chronic illness, and site of data collection. The second section assessed the ability of participants to recognize 13 myths around CC causation as being incorrect. All but one of the examined myths were adopted from the original CAM-MYCS [9]. The item ‘eating burnt food’ was deemed important to be added [28].

The Arabic questionnaire was modified for the purposes of this study. The original questions of the CAM-MYCS with correct/incorrect/unsure responses were modified into 5-point Likert scale questions (1 = strongly disagree, 2 = disagree, 3 = not sure, 4 = agree, 5 = strongly agree) to minimize the possibility of participants answering questions at random. Meanwhile, the participants’ responses were then converted to correct/incorrect responses, where responses with ‘strongly disagree’ or ‘disagree’ were considered correct and all other responses were considered incorrect.

Eligible participants were invited to a face-to-face interview to complete the questionnaire with the presence of a data collector. Data were collected using Kobo Toolbox, a reliable and user-friendly application that can be utilized on smartphones [29].

### Statistical analysis

Descriptive statistics were used to describe participant characteristics. Categorical variables were described using frequencies and percentages, whereas continuous variables, which were non-normally distributed, were described using the median and interquartile range (IQR). The Kruskal–Wallis test was used to compare baseline characteristics of participants recruited from the Gaza Strip and those recruited from the WBJ if the characteristic was continuous, while Pearson's Chi-square test was used if the characteristic was categorical.

Age was categorized into three groups to reflect the age-associated risk of CC: 18–20 years, 21–40 years (at-risk group), and ≥ 41 years [30]. Since the minimum wage in Palestine is 1450 NIS (about $450) [31], the monthly income was also categorized into two categories: < 1450 NIS and ≥ 1450 NIS.

The examined myths around causes of CC were classified into food-related and food-unrelated causes. Frequencies and percentages were used to describe the recognition of each myth and Pearson's Chi-Square test was used for comparisons between participants from the Gaza Strip versus those from the WBJ.

Multivariable logistic regression analyses were utilized to examine the association between participant characteristics and recognizing each myth around CC causation as being incorrect. The multivariable analyses adjusted for age-group, educational level, employment status, monthly income, marital status, place of residency, having a chronic disease, knowing someone with cancer, and site of data collection. This model was determined a priori based on previous studies [9, 10, 13–15, 32, 33].

Awareness of the investigated myths about causes of CC was assessed utilizing a scoring system, which had also been used in previous studies [13–15, 19–27, 34]. The participant received one point for each correctly recognized myth. The total score (ranging from 0 to 13) was calculated and categorized into three categories based on the number of myths recognized as being incorrect: poor (0 to 4), fair (5 to 9), and good awareness (10 to 13). The ability to recognize myths around CC causation to be incorrect was compared between participants from the Gaza Strip and those from the WBJ using Pearson's Chi-Square test. A multivariable logistic regression analysis was utilized to examine the association between participant characteristics and displaying good awareness. The same aforementioned multivariable model was used.

Missing data were hypothesized to be missed completely at random and thus, complete case analysis was utilized to handle them. Data were analyzed using Stata software version 17.0 (StataCorp, College Station, Texas, United States).

## Results

### Participant characteristics

Out of 8086 approached, a total of 7223 participants agreed and completed the questionnaire (response rate = 89.3%). A total of 7058 questionnaires were included in the final analysis (30 did not meet inclusion criteria and 135 had missing values); 2655 were from the Gaza Strip and 4403 from the WBJ. Participants recruited from the Gaza Strip were younger, earned lower monthly income, and had fewer chronic diseases than those recruited from the WBJ (Table 1).

**Table 1:** Characteristics of study participants

Characteristic	Total (*n* = 7058)	Gaza Strip (*n* = 2655)	WBJ (*n* = 4403)	*p*-value
**Age**, median [IQR]	32.0 [24.0, 42.0]	30.0 [24.0, 39.0]	33.0 [24.0, 44.0]	< 0.001
**Age group**, n (%)				
18 to 20	756 (10.7)	249 (9.4)	507 (11.5)	< 0.001
21 to 40	4331(61.4)	1809 (68.1)	2522 (57.3)	
41 or older	1971 (27.9)	597 (22.5)	1374 (31.2)	
**Educational level**, n (%)				
Secondary or below	3893 (55.2)	1497 (56.4)	2396 (54.4)	0.11
Above secondary	3165 (44.8)	1158 (43.6)	2007 (45.6)	
**Occupation**, n (%)				
Housewife	4647 (65.8)	2008 (75.6)	2639 (59.9)	< 0.001
Employed	1476 (20.9)	348 (13.1)	1128 (25.6)	
Retired	69 (1.0)	11 (0.5)	58 (1.4)	
Student	866 (12.3)	288 (10.8)	578 (13.1)	
**Monthly income ≥ 1450 NIS**, n (%)	4666 (66.1)	693 (26.1)	3973 (90.2)	< 0.001
**Marital status**, n (%)				
Single	1657 (23.5)	527 (19.8)	1130 (25.7)	< 0.001
Married	5058 (71.6)	(76.3)	3033 (68.8)	
Divorced/Widowed	343 (4.9)	103 (3.9)	240 (5.5)	
**Having a chronic disease**, n (%)	1397 (19.8)	417 (15.7)	980 (22.3)	< 0.001
**Knowing someone with cancer**, n (%)	4083 (57.9)	1483 (55.9)	2600 (59.1)	0.009
**Site of data collection**, n (%)				
Public spaces	2.695 (38.2)	863 (32.5)	1832 (41.6)	< 0.001
Hospitals	1890 (26.7)	642 (24.2)	1248 (28.4)	
Primary healthcare centers	2473 (35.1)	1150 (43.3)	1323 (30.0)	

*n* Number of participants, *IQR* Interquartile range, *WBJ* West Bank and Jerusalem

### Recognition of myths around CC causation to be incorrect

In general, the myths around CC causation unrelated to food were more commonly recognized to be incorrect by participants than those related to food. The food-unrelated myth around CC causation that was most commonly recognized was ‘having a physical trauma’ (*n* = 3714, 52.6%), whereas the one least commonly recognized was ‘using mobile phones’ (*n* = 2238, 31.7%) (Table 2). The food-related myth around CC causation that was most commonly recognized was ‘drinking from plastic bottles’ (*n* = 2708, 38.4%), whereas the least recognized was ‘eating food containing additives’ (*n* = 1118, 15.8%).

**Table 2:** Summary of the assessment of public beliefs in mythical causes of cervical cancer

Myth	Total (*n* = 7058) *n* (%)	Gaza Strip (*n* = 2655) *n* (%)	WBJ (*n* = 4403) *n* (%)	*p*–value
**Food-related**				
Drinking from plastic bottles	2708 (38.4)	1125 (42.4)	1583 (36.0)	< 0.001
Eating burnt food (e.g., bread or barbeque)	2272 (32.2)	762 (28.7)	1510 (34.3)	< 0.001
Eating food containing artificial sweeteners (e.g., saccharine)	1938 (27.5)	759 (28.6)	1179 (26.8)	0.10
Using microwave ovens	1484 (21.0)	646 (24.3)	838 (19.0)	< 0.001
Eating genetically modified food (e.g., hybrid vegetables)	1246 (17.7)	531 (20.0)	715 (16.2)	< 0.001
Eating food containing additives	1118 (15.8)	412 (15.5)	706 (16.0)	0.56
**Others**				
Having a physical trauma	3714 (52.6)	1415 (53.3)	2299 (52.2)	0.38
Using aerosol containers	3428 (48.6)	1353 (51.0)	2075 (47.1)	0.002
Using cleaning products	2900 (41.1)	1203 (45.3)	1697 (38.5)	< 0.001
Feeling stressed	2621 (37.1)	1090 (41.1)	1531 (34.8)	< 0.001
Living near power lines	2349 (33.3)	991 (37.3)	1358 (30.8)	< 0.001
Exposure to electromagnetic frequencies (e.g., Wi-Fi and Radio/TV frequencies)	2253 (31.9)	925 (34.8)	1328 (30.2)	< 0.001
Using mobile phones	2238 (31.7)	988 (37.2)	1250 (28.4)	< 0.001

*n* Number of participants. *WBJ* West Bank and Jerusalem

### The ability to recognize myths around CC causation and its associated factors

Only 575 participants (8.1%) displayed good awareness and were able to recognize 10 or more myths around CC causes to be incorrect (Table 3). Participants from the Gaza Strip were more likely than participants from the WBJ to display good awareness of the examined myths to be incorrect (10.3% vs. 6.8%). Besides living in the WBJ, being married, widowed or divorced, knowing someone with cancer, and visiting hospitals or primary healthcare centers were all associated with lower likelihood to display good awareness and recognize myths around CC causation to be incorrect (Table 4).

**Table 3:** Recognition of mythical causes of cervical cancer among study participants

**Level**	**Total** *n* (%)	**Gaza Strip** *n* (%)	**WBJ** *n* (%)	***p*–value**
**Poor** (0–4 myths)	4044 (57.3)	1415 (53.3)	2629 (59.7)	< 0.001
**Fair** (5–9 myths)	2439 (34.6)	966 (36.4)	1473 (33.5)	
**Good** (10–13 myths)	575 (8.1)	274 (10.3)	301 (6.8)	

*n* Number of participants, *WBJ* = West Bank and Jerusalem

**Table 4:** Bivariable and multivariable logistic regression analyzing factors associated with having good recognition of the mythical causes of cervical cancer

Characteristic	Good knowledge			
	**COR (95% CI)**	***p*-value**	AOR (95% CI)*	***p*-value**
**Age group**				
18 to 20	Ref	Ref	Ref	Ref
21 to 40	0.63 (0.50- 0.80)	< 0.001	0.84 (0.61- 1.15)	0.27
41 or older	0.48 (0.36- 0.63)	< 0.001	0.74 (0.50- 1.08)	0.12
**Educational level**				
Secondary or below	Ref	Ref	Ref	Ref
Post-secondary	1.19 (1.01- 1.41)	0.043	0.92 (0.75- 1.13)	0.42
**Occupation**				
Housewife	Ref	Ref	Ref	Ref
Employed	1.21 (0.98- 1.50)	0.07	0.97 (0.75- 1.26)	0.83
Retired	0.58 (0.18- 1.86)	0.36	0.60 (0.18- 1.98)	0.40
Student	1.80 (1.43- 2.27)	< 0.001	0.82 (0.58- 1.15)	0.25
**Monthly income**				
< 1450 NIS	Ref	Ref	Ref	Ref
≥ 1450 NIS	0.81 (0.68- 0.96)	0.016	1.00 (0.79- 1.27)	0.99
**Marital status**				
Single	Ref	Ref	Ref	Ref
Married	0.52 (0.43- 0.62)	< 0.001	0.62 (0.48- 0.79)	< 0.001
Divorced/Widowed	0.41 (0.25- 0.67)	< 0.001	0.46 (0.27- 0.78)	0.004
**Residency**				
Gaza Strip	Ref	Ref	Ref	Ref
WBJ	0.64 (0.54- 0.76)	< 0.001	0.58 (0.46- 0.72)	< 0.001
**Having a chronic disease**				
No	Ref	Ref	Ref	Ref
Yes	0.80 (0.64- 1.00)	0.052	1.10 (0.85- 1.43)	0.47
**Knowing someone with cancer**				
No	Ref	Ref	Ref	Ref
Yes	0.67 (0.57- 0.80)	< 0.001	0.66 (0.55- 0.79)	< 0.001
**Site of data collection**				
Public Spaces	Ref	Ref	Ref	Ref
Hospitals	0.49 (0.40- 0.61)	< 0.001	0.53 (0.42- 0.67)	< 0.001
Primary healthcare centers	0.40 (0.33- 0.50)	< 0.001	0.39 (0.31- 0.49)	< 0.001

*COR* Crude odds ratio, *AOR* Adjusted odds ratio, *CI* Confidence interval, *WBJ* West Bank and Jerusalem

^*^Adjusted for age-group, educational level, occupation, monthly income, marital status, residency, having a chronic disease, knowing someone with cancer, and site of data collection

### Association between recognizing CC food-related mythical causes and participant characteristics

Participants recruited from hospitals or primary healthcare centers were less likely than participants recruited from public spaces to recognize all CC food-related myths (Table 5). In addition, participants ≥ 20 years were less likely than younger participants to recognize all examined food-related myths around CC causation to be incorrect, except ‘using microwave ovens’, where no associated difference was found. Similarly, married participants were less likely than single participants to recognize all examined food-related myths around CC causes to be incorrect, except ‘eating food containing artificial sweeteners, where no associated difference was observed. Moreover, participants who knew someone with cancer were less likely than participants who did not to recognize all CC food-related myths except for ‘eating burnt food’, where no associated difference was found. Finally, participants from the WBJ were less likely than participants from the Gaza Strip to recognize all CC food-related myths to be incorrect, except ‘eating burnt food’ and ‘eating food containing additives’, where no associated differences were found. There was no association between education level and recognizing all CC food-related myths except ‘eating genetically modified food’, which participants with higher education were less likely to recognize it to be incorrect.

**Table 5:** Multivariable logistic regression analyzing factors associated with the recognition of each mythical food-related cause of cervical cancer

Characteristic	Drinking from plastic bottles		Eating burnt food		Eating food containing artificial sweeteners		Using microwave ovens	
	AOR (95% CI)*	***p*-value**	AOR (95% CI)*	***p*-value**	AOR (95% CI)*	***p*-value**	AOR (95% CI)*	***p*-value**
**Age group**								
18 to 20	Ref	Ref	Ref	Ref	Ref	Ref	Ref	Ref
21 to 40	0.60 (0.49- 0.73)	< 0.001	0.84 (0.68- 1.02)	0.08	0.67 (0.54- 0.82)	< 0.001	0.85 (0.68- 1.06)	0.16
41 or older	0.43 (0.34- 0.54)	< 0.001	0.66 (0.52- 0.83)	< 0.001	0.62 (0.49- 0.79)	< 0.001	0.80 (0.62- 1.04)	0.10
**Educational level**								
Secondary or below	Ref	Ref	Ref	Ref	Ref	Ref	Ref	Ref
Post–secondary	1.02 (0.91- 1.15)	0.70	1.08 (0.95- 1.21)	0.23	0.98 (0.87- 1.11)	0.78	1.05 (0.92- 1.21)	0.45
**Occupation**								
Unemployed/housewife	Ref	Ref	Ref	Ref	Ref	Ref	Ref	Ref
Employed	0.86 (0.74- 0.99)	0.037	0.95 (0.82- 1.11)	0.53	0.99 (0.85- 1.16)	0.91	1.06 (0.89- 1.25)	0.54
Retired	0.88 (0.51- 1.51)	0.64	1.05 (0.61- 1.79)	0.86	1.24 (0.72- 2.16)	0.44	1.19 (0.65- 2.19)	0.57
Student	0.93 (0.75- 1.15)	0.49	1.10 (0.89- 1.37)	0.38	1.00 (0.80- 1.26)	0.97	0.97 (0.76- 1.23)	0.780.78
**Monthly income**								
< 1450 NIS	Ref	Ref	Ref	Ref	Ref	Ref	Ref	Ref
≥ 1450 NIS	1.14 (0.99- 1.31)	0.08	1.13 (0.97- 1.31)	0.11	1.07 (0.92- 1.25)	0.40	0.99 (0.84- 1.17)	0.95
**Marital status**								
Single	Ref	Ref	Ref	Ref	Ref	Ref	Ref	Ref
Married	0.74 (0.64- 0.86)	< 0.001	0.82 (0.71- 0.96)	0.012	0.91 (0.78- 1.07)	0.27	0.69 (0.58- 0.82)	< 0.001
Divorced/Widowed	0.58 (0.44- 0.76)	< 0.001	0.86 (0.66- 1.13)	0.29	0.95 (0.71- 1.26)	0.71	0.63 (0.46- 0.87)	0.005
**Residency**								
Gaza Strip	Ref	Ref	Ref	Ref	Ref	Ref	Ref	Ref
WBJ	0.66 (0.58- 0.76)	< 0.001	1.14 (0.99- 1.31)	0.07	0.78 (0.67- 0.90)	< 0.001	0.70 (0.60- 0.82)	< 0.001
**Having a chronic disease**								
No	Ref	Ref	Ref	Ref	Ref	Ref	Ref	Ref
Yes	1.16 (1.01- 1.33)	0.041	1.10 (0.95- 1.27)	0.21	1.03 (0.88- 1.20)	0.70	0.95 (0.80- 1.13)	0.58
**Knowing someone with cancer**								
No	Ref	Ref	Ref	Ref	Ref	Ref	Ref	Ref
Yes	0.88 (0.80- 0.98)	0.017	0.92 (0.83- 1.02)	0.10	0.84 (0.76- 0.94)	0.002	0.81 (0.72- 0.92)	0.001
**Site of data collection**								
Public Spaces	Ref	Ref	Ref	Ref	Ref	Ref	Ref	Ref
Hospitals	0.65 (0.57- 0.74)	< 0.001	0.67 (0.58- 0.76)	< 0.001	0.59 (0.51- 0.68)	< 0.001	0.68 (0.58- 0.80)	< 0.001
Primary healthcare centers	0.52 (0.46- 0.59)	< 0.001	0.56 (0.49- 0.63)	< 0.001	0.37 (0.33- 0.43)	< 0.001	0.75 (0.65- 0.87)	< 0.001
**Characteristic**		Eating genetically modified food			Eating food containing additives			
		AOR (95% CI)*		**p-value**	AOR (95% CI)*		**p-value**	
**Age group**								
18 to 20		Ref		Ref	Ref		Ref	
21 to 40		0.76 (0.60- 0.96)		0.024	0.76 (0.60- 0.96)		0.023	
41 or older		0.55 (0.42- 0.73)		< 0.001	0.56 (0.42- 0.75)		< 0.001	
**Educational level**								
Secondary or below		Ref		Ref	Ref		Ref	
Post–secondary		0.82 (0.71- 0.95)		0.008	1.05 (0.90- 1.22)		0.54	
**Occupation**								
Unemployed/housewife		Ref		Ref	Ref		Ref	
Employed		0.88 (0.73- 1.06)		0.19	0.87 (0.72- 1.06)		0.16	
Retired		0.90 (0.43- 1.87)		0.77	0.66 (0.31- 1.44)		0.30	
Student		0.81 (0.62- 1.05)		0.11	0.82 (0.63- 1.07)		0.15	
**Monthly income**								
< 1450 NIS		Ref		Ref	Ref		Ref	
≥ 1450 NIS		1.11 (0.93- 1.32)		0.25	1.15 (0.96- 1.39)		0.13	
**Marital status**								
Single		Ref		Ref	Ref		Ref	
Married		0.75 (0.62- 0.90)		0.002	0.64 (0.53–0.78)		< 0.001	
Divorced/Widowed		0.60 (0.42- 0.87)		0.007	0.68 (0.47- 0.97)		0.033	
**Residency**								
Gaza Strip		Ref		Ref	Ref		Ref	
WBJ		0.72 (0.61- 0.85)		< 0.001	0.92 (0.77- 1.10)		0.35	
**Having a chronic disease**								
No		Ref		Ref	Ref		Ref	
Yes		0.98 (0.82- 1.18)		0.85	1.02 (0.84- 1.24)		0.83	
**Knowing someone with cancer**								
No		Ref		Ref	Ref		Ref	
Yes		0.73 (0.65- 0.83)		< 0.001	0.72 (0.63- 0.82)		< 0.001	
**Site of data collection**								
Public Spaces		Ref		Ref	Ref		Ref	
Hospitals		0.81 (0.69- 0.96)		0.013	0.82 (0.69- 0.97)		0.020	
Primary healthcare centers		0.67 (0.57- 0.78)		< 0.001	0.61 (0.51- 0.72)		< 0.001	

*AOR* Adjusted odds ratio, *CI* Confidence interval, *WBJ* West Bank and Jerusalem

^*^Adjusted for age-group, educational level, occupation, monthly income, marital status, residency, having a chronic disease, knowing someone with cancer, and site of data collection

### Association between recognition of food-unrelated myths around CC causation and participant characteristics

Participants from the WBJ were less likely than participants from the Gaza Strip to recognize all examined myths around CC causes, unrelated to food, to be incorrect (Table 6). In addition, participants ≥ 40 years were less likely than younger participants to recognize all examined food-unrelated myths around CC causes, except ‘having a physical trauma’, where no associated difference was found. Participants recruited from hospitals or primary healthcare centers were less likely than participants recruited from public spaces to recognize five out of the seven food-unrelated myths.

**Table 6:** Multivariable logistic regression analyzing factors associated with the recognition of each other mythical cause of cervical cancer

Characteristic	Having a physical trauma		Using aerosol containers		Using cleaning products		Feeling stressed	
	AOR (95% CI)*	***p*-value**	AOR (95% CI)*	***p-*value**	AOR (95% CI)*	***p*-value**	AOR (95% CI)*	***p*-value**
**Age group**								
18 to 20	Ref	Ref	Ref	Ref	Ref	Ref	Ref	Ref
21 to 40	1.07 (0.88- 1.31)	0.47	0.64 (0.53- 0.78)	< 0.001	0.83 (0.68- 1.00)	0.06	0.80 (0.65- 0.97)	0.024
41 or older	0.96 (0.77- 1.19)	0.69	0.47 (0.37- 0.59)	< 0.001	0.57 (0.45- 0.71)	< 0.001	0.70 (0.56- 0.88)	0.002
**Educational level**								
Secondary or below	Ref	Ref	Ref	Ref	Ref	Ref	Ref	Ref
Post–secondary	1.09 (0.98- 1.22)	0.11	1.04 (0.93- 1.16)	0.54	0.84 (0.75- 0.94)	0.003	0.95 (0.84- 1.06)	0.34
**Occupation**								
Unemployed/housewife	Ref	Ref	Ref	Ref	Ref	Ref	Ref	Ref
Employed	0.92 (0.80- 1.06)	0.27	0.83 (0.72- 0.95)	0.009	0.96 (0.83- 1.10)	0.53	0.85 (0.73- 0.98)	0.028
Retired	0.52 (0.31- 0.87)	0.012	0.71 (0.42- 1.20)	0.20	0.84 (0.49- 1.43)	0.52	0.82 (0.48- 1.41)	0.47
Student	0.96 (0.78- 1.18)	0.68	0.92 (0.74- 1.14)	0.44	0.89 (0.72- 1.10)	0.29	0.93 (0.75- 1.15)	0.48
**Monthly income**								
< 1450 NIS	Ref	Ref	Ref	Ref	Ref	Ref	Ref	Ref
≥ 1450 NIS	1.30 (1.13- 1.49)	< 0.001	1.42 (1.24- 1.64)	< 0.001	1.18 (1.02- 1.35)	0.022	1.24 (1.07- 1.42)	0.003
**Marital status**								
Single	Ref	Ref	Ref	Ref	Ref	Ref	Ref	Ref
Married	1.09 (0.94- 1.26)	0.26	0.84 (0.72- 0.97)	0.019	0.82 (0.71- 0.96)	0.011	0.96 (0.83- 1.12)	0.62
Divorced/Widowed	0.91(0.71- 1.18)	0.48	0.76 (0.58- 0.98)	0.033	0.84 (0.65- 1.09)	0.20	0.84 (0.64- 1.10)	0.20
**Residency**								
Gaza Strip	Ref	Ref	Ref	Ref	Ref	Ref	Ref	Ref
WBJ	0.81 (0.71- 0.93)	0.002	0.70 (0.62- 0.80)	< 0.001	0.69 (0.60- 0.79)	< 0.001	0.67 (0.59- 0.77)	< 0.001
**Having a chronic disease**								
No	Ref	Ref	Ref	Ref	Ref	Ref	Ref	Ref
Yes	1.00 (0.88- 1.14)	0.97	1.10 (0.97- 1.26)	0.15	1.28 (1.12- 1.47)	< 0.001	1.09 (0.95- 1.25)	0.22
**Knowing someone with cancer**								
No	Ref	Ref	Ref	Ref	Ref	Ref	Ref	Ref
Yes	0.96 (0.87- 1.05)	0.38	1.02 (0.93- 1.13)	0.63	0.89 (0.81- 0.99)	0.025	0.84 (0.76- 0.92)	< 0.001
**Site of data collection**								
Public Spaces	Ref	Ref	Ref	Ref	Ref	Ref	Ref	Ref
Hospitals	1.10 (0.97- 1.25)	0.14	0.72 (0.64- 0.82)	< 0.001	0.86 (0.76- 0.98)	0.020	0.82 (0.72- 0.93)	0.002
Primary healthcare centers	0.82 (0.73- 0.92)	0.001	0.92 (0.82- 1.03)	0.16	0.93 (0.82- 1.04)	0.20	0.80 (0.71- 0.90)	< 0.001
**Characteristic**	Living near power lines		Exposure to electromagnetic frequencies		Using mobile phones			
	AOR (95% CI)*	***p*-value**	AOR (95% CI)*	***p*-value**	AOR (95% CI)*		***p*-value**	
**Age group**								
18 to 20	Ref	Ref	Ref	Ref	Ref		Ref	
21 to 40	0.61 (0.50- 0.75)	< 0.001	0.76 (0.62- 0.94)	0.010	0.60 (0.49- 0.73)		< 0.001	
41 or older	0.48 (0.38- 0.60)	< 0.001	0.70 (0.56- 0.89)	0.003	0.48 (0.38- 0.60)		< 0.001	
**Educational level**								
Secondary or below	Ref	Ref	Ref	Ref	Ref		Ref	
Post–secondary	1.11 (0.99- 1.25)	0.07	0.94 (0.83- 1.06)	0.31	1.16 (1.03- 1.31)		0.013	
**Occupation**								
Unemployed/housewife	Ref	Ref	Ref	Ref	Ref		Ref	
Employed	0.98 (0.84- 1.13)	0.75	0.91 (0.78- 1.06)	0.24	1.05 (0.90- 1.22)		0.53	
Retired	0.79 (0.44- 1.41)	0.42	0.64 (0.36- 1.15)	0.14	0.94 (0.53- 1.63)		0.81	
Student	0.95 (0.76- 1.17)	0.62	0.74 (0.59- 0.93)	0.009	0.92 (0.74- 1.15)		0.48	
**Monthly income**								
< 1450 NIS	Ref	Ref	Ref	Ref	Ref		Ref	
≥ 1450 NIS	1.00 (0.86- 1.15)	0.96	0.91 (0.78- 1.05)	0.19	1.04 (0.90- 1.21)		0.59	
**Marital status**								
Single	Ref	Ref	Ref	Ref	Ref		Ref	
Married	0.89 (0.77- 1.04)	0.15	0.85 (0.73- 0.10)	0.046	0.73 (0.63- 0.85)		< 0.001	
Divorced/Widowed	0.75 (0.56- 0.99)	0.045	0.76 (0.58- 1.00)	0.054	0.72 (0.55- 0.96)		0.023	
**Residency**								
Gaza Strip	Ref	Ref	Ref	Ref	Ref		Ref	
WBJ	0.75 (0.65- 0.86)	< 0.001	0.82 (0.71- 0.94)	0.004	0.59 (0.51- 0.68)		< 0.001	
**Having a chronic disease**								
No	Ref	Ref	Ref	Ref	Ref		Ref	
Yes	0.98 (0.85- 1.13)	0.77	1.06 (0.92- 1.23)	0.40	1.13 (0.98- 1.31)		0.10	
**Knowing someone with cancer**								
No	Ref	Ref	Ref	Ref	Ref		Ref	
Yes	0.91 (0.82- 1.01)	0.07	0.79 (0.72- 0.88)	< 0.001	0.80 (0.72- 0.88)		< 0.001	
**Site of data collection**								
Public Spaces	Ref	Ref	Ref	Ref	Ref		Ref	
Hospitals	0.82 (0.72- 0.94)	0.004	1.02 (0.89- 1.16)	0.80	0.69 (0.60- 0.79)		< 0.001	
Primary healthcare centers	0.85 (0.75- 0.96)	0.009	0.59 (0.52- 0.68)	< 0.001	0.58 (0.51- 0.66)		< 0.001	

*AOR* Adjusted odds ratio, *CI* Confidence interval, *WBJ* West Bank and Jerusalem

^*^Adjusted for age-group, educational level, occupation, monthly income, marital status, residency, having a chronic disease, knowing someone with cancer, and site of data collection

Participants who knew someone with cancer were less likely than participants who did not to recognize four out of the seven food-unrelated myths around CC causation to be incorrect. In contrast, participants with higher monthly income were more likely to recognize the four most frequently reported food un-related myths around CC causes, namely ‘having a physical trauma’, ‘using aerosol containers’, ‘using cleaning products’, and ‘feeling stressed’. There was no association between education level and recognition of five out of the seven food-related myths of CC causes.

## Discussion

Dispelling myths related to CC causation in Palestine holds paramount importance in promoting informed health practices and preventive measures. This study investigated the ability of Palestinian women to recognize myths around CC causes to be incorrect and explore the factors associated with good recognition. The study findings indicated that only 575 participants (8.1%) showed a good ability to recognize the examined myths, defined as being able to identify more than nine out of 13 myths around CC causation to be incorrect. Compared to WBJ participants, women from the Gaza Strip were more likely to recognize the myths to be incorrect (10.3% vs. 6.8%). Aside from living in the WBJ, being married, widowed, or divorced, knowing someone with cancer, and visiting hospitals or primary healthcare centers were all associated with lesser abilities to recognize myths around CC causes to be incorrect.

This study showed low awareness and highlighted misconceptions of CC mythical causes, in concordance with previous studies conducted in the United Kingdom and China [10, 35]. Women frequently linked certain environmental and lifestyle factors—which have no significant impact on the development of the disease —with higher risks to develop CC. These misconceptions may mislead women and impact their health beliefs, by distracting focus on such fictitious risk factors, instead of making healthy lifestyle adjustments that could lower their risk of developing cancer [36, 37]. Lifestyle changes alone could prevent between one-third to one-half of cancer cases [37, 38]. Better understanding of the environmental and lifestyle variables that increase risks of developing cancer may encourage behaviours to reduce such risks [37, 38]. It is essential to effectively raise public awareness of myths around CC causes, which may impact their attitudes and behaviours and target effective lifestyle changes by reducing distractions [9, 10]. Particularly in low- and middle-income settings, like Palestine, which lacks HPV vaccination and CC screening programs, such awareness is important [13–15].

The most important risk factor for CC is infection with high-grade HPV subtypes [39, 40]. In Palestine, HPV infection has been identified in 89% of CC specimens, 68% of pregnant women, and 25% of non-pregnant women [41]. Awareness of women regarding the association between HPV infection and CC is pivotal for encouraging participation in CC screening programs and fostering acceptance of vaccination. This significance is particularly highlighted by the success of HPV immunization programs in diminishing the morbidity and mortality rates linked with CC [42].

Preventing CC among Palestinian women necessitates a nuanced approach that considers the cultural, social, and religious aspects shaping their healthcare practices. Preventive practices, such as regular screenings through Pap smears and HPV vaccinations, have been shown to reduce the morbidity and mortality associated with CC [42–44]. However, in addition to the lack of CC screening programs and HPV vaccination as part of the national health policy, numerous barriers may exist and be intertwined with cultural norms and religious beliefs. Women may be discouraged from obtaining preventative care because of cultural taboos around talking about reproductive health especially if they were unmarried. Moreover, important information concerning CC and potential preventive strategies can be hindered by social factors, such as low awareness and educational opportunities. Religious considerations, particularly modesty concerns, may also influence women's hesitancy to undergo screening if it becomes available. Collaborative efforts are required to bridge these gaps, involving community leaders, healthcare providers, and educational institutions. Culturally sensitive and linguistically appropriate health campaigns can dispel misconceptions, emphasizing the importance of preventive practices while respecting the unique cultural and religious contexts of Palestinian women.

Similar to this study, Sherman and colleagues reported a significant belief in the mythical causes of CC, such as stress (14%), living near power lines (7%), pollution (5%), and food additives (5%) [45]. Another study conducted in Iran found that some women thought that stress might contribute to CC. As a result, women used various techniques to combat their stress, such as relaxing, listening to music, and exercising [46]. The propagation of such misconceptions might be a consequence of social media use. Social media has accelerated the spread of myths and misinformation, which may have negatively impacted women's trust in health information and may have made it more challenging for women to distinguish the actual causes of cancer from the myths that are presented incorrectly as causes for cancer [47–50]. Consequently, this might cause women to disregard evidence-based public health measures [51]. An important part of the role of healthcare providers is to guide women towards reliable sources of health information and, thus, increase their health literacy. This is especially important given that participants visiting healthcare facilities were less likely to recognize myths of CC causes to be incorrect.

A study conducted in six public schools in Kenya found that schools and mass media were the primary sources of information about CC [52]. School curricula should encourage young women to seek health information from evidence-based sources, and to nurture awareness of possible misinformation on the internet and social media. This study showed no difference between participants with higher education and those with lower educational achievements in terms of recognizing most examined myths to be incorrect. This highlights the need for inclusion of CC mythical causes in the school curricula as well as interventions to increase health literacy in primary healthcare centers to target a broad section of the population. An intervention of anti-CC teaching targeting high school students in Nigeria was effective for increasing awareness related to myths and appeared to be sustained if engagement action was maintained over time [53]. It is possible for newly acquired knowledge or attitudes to decline over time as a result of ongoing external stimuli or beliefs [54]. Ifediora and colleagues considered CC myths could be hard to tackle, unless the real reasons for these myths can be unraveled [53]. Therefore, policymakers should ensure extra efforts to maintain targeting myths during health campaigns [53].

### Future directions

This study highlighted some myths and misconceptions about CC causation among Palestinian women. These misconceptions might be corrected using educational interventions targeting the public through mass media, social media and school curricula. Furthermore, healthcare institutions should play a role in increasing the public awareness since a high percentage of the population living in Palestine may have no access to online sources [55] and participants attending these institutions seem to have lower awareness. By addressing and correcting these myths, healthcare initiatives can promote public understanding of the actual risk factors associated with CC like HPV infection. This, in turn, may enhance timely engagement in CC screening programs and may facilitate acceptance of preventive measures like HPV vaccinations when they become available. Additionally, dispelling myths contributes to reducing the stigma surrounding CC, fostering an environment that encourages open discussions about reproductive health and empowers women to make informed decisions about their well-being.

### Limitations

The use of a convenience sample may impact the generalizability of the results, but this may have been mitigated by the large sample size, data collection from various geographic locations, and high response rate. A further limitation could be the exclusion of participants with medical backgrounds who presumably have good awareness levels of CC mythical causes. Nonetheless, their exclusion was intended to increase the relevance of the study as a measure of the public awareness. Finally, there could exist some other myths related to HPV vaccination and CC screening that might be interesting to assess their prevalence in the Palestinian community. However, the focus of this study was to examine CC causation myths that were included in the validated CAM-MYCS [9].

## Conclusions

Only 8.1% of the study participants could identify 10 or more out of the 13 examined myths around CC causes to be incorrect. Factors associated with lower likelihood to recognize myths around CC causation to be incorrect included living in the WBJ, being married, widowed or divorced, knowing someone with cancer, and visiting hospitals or primary healthcare centers. CC mythical causes unrelated to food were recognized more frequently than those related to food. Overall, this study demonstrates a necessity for a national strategy to generate a better understanding of CC causes and dispel the widely held misconceptions. Integrating social media platforms and school-based health education could be an effective strategy to promote community knowledge, health literacy and support health promotion initiatives.

### Supplementary Information

**Additional file 1. **

## Data Availability

The dataset used and analyzed during the current study will be available by the corresponding author upon reasonable request.

## Abbreviations

CC: Cervical cancer
HPV: Human papillomavirus
WBJ: West Bank and Jerusalem
CI: Confidence interval
OR: Odds ratio
