# Supplementary material for: Myths and common misbeliefs about cervical cancer causation among Palestinian women: a national cross-sectional study
Source: BMC Public Health. 2024 Jan 16;24:189. doi: 10.1186/s12889-024-17733-5 (PMC10790379; doi:10.1186/s12889-024-17733-5)
Supplement: Supplementary file 1 — Additional file 1. [file 12889_2024_17733_MOESM1_ESM.docx]

**Awareness of Cervical Cancer Causation Myths Questionnaire**

Serial number: ………. Location: …………. Governorate: ……….

1. **Sociodemographic Data**

- Age: …….. years
- Marital status: 🞏 Single 🞏 Married 🞏 Divorced 🞏 Widowed
- Highest level of education: 🞏 Illiterate 🞏 Primary 🞏 Prep 🞏 Secondary

🞏 Diploma 🞏 Bachelor degree 🞏 Postgraduate

- Occupation: 🞏 Unemployed/Housewife 🞏 Employed 🞏 Retired 🞏 Student
- Monthly income (NIS): ………………
- Do you have any chronic disease? 🞏 No 🞏 Yes
- Have any of your family members or close friends had cancer? 🞏 No 🞏 Yes

**2. Recognition of Cervical Cancer Causation Myths**

| Table (1): The following practices may or may not cause cervical cancer. We are interested in your opinion. | | | | | |
| --- | --- | --- | --- | --- | --- |
| Belief | **1= Strongly Disagree** | **2= Disagree** | **3= Not Sure** | **4= Agree** | **5= Strongly agree** |
| 1. Drinking from plastic bottles |  |  |  |  |  |
| 1. Eating food containing artificial sweeteners |  |  |  |  |  |
| 1. Eating genetically modified food |  |  |  |  |  |
| 1. Eating food containing additives |  |  |  |  |  |
| 1. Using microwave ovens |  |  |  |  |  |
| 1. Using aerosol containers |  |  |  |  |  |
| 1. Using mobile phones |  |  |  |  |  |
| 1. Using cleaning products |  |  |  |  |  |
| 1. Living near power lines |  |  |  |  |  |
| 1. Feeling stressed |  |  |  |  |  |
| 1. Having a physical trauma |  |  |  |  |  |
| 1. Exposure to electromagnetic frequencies (e.g, Wi-Fi and Radio/TV frequencies) |  |  |  |  |  |
| 1. Eating burnt food (e.g., bread or barbeque) |  |  |  |  |  |
